# Tumour burden score combined with albumin‐to‐alkaline phosphatase ratio predicts prognosis in patients with intrahepatic cholangiocarcinoma

**DOI:** 10.1111/jcmm.18530

**Published:** 2024-07-03

**Authors:** Sheng Wang, Maoyun Liu, Lei Xiang, Haizhou Qiu, Luo Cheng, Zuotian Huang, Tao Wen, Wenyuan Xie, Sipeng Li, Cheng Zhang, Genshu Wang, Hui Li, Dewei Li

**Affiliations:** ^1^ School of Medicine, Chongqing University Chongqing University Cancer Hospital Chongqing China; ^2^ Department of Hepatobiliary Pancreatic Tumor Center Chongqing University Cancer Hospital Chongqing China; ^3^ Department of Liver Surgery, West China Hospital Sichuan University Chengdu China; ^4^ Department of Liver Transplantation, State Key Laboratory of Traditional Chinese Medicine Syndrome, Guangdong Provincial Hospital of Chinese Medicine The Second Affiliated Hospital of Guangzhou University of Chinese Medicine Guangzhou China

**Keywords:** albumin‐to‐alkaline phosphatase ratio, curative resection, intrahepatic cholangiocarcinoma, long‐term outcome, tumour burden score

## Abstract

Tumour morphology (tumour burden score (TBS)) and liver function (albumin‐to‐alkaline phosphatase ratio (AAPR)) have been shown to correlate with outcomes in intrahepatic cholangiocarcinoma (ICC). This study aimed to evaluate the combined predictive effect of TBS and AAPR on survival outcomes in ICC patients. We conducted a retrospective analysis using a multicentre database of ICC patients who underwent curative surgery from 2011 to 2018. The Kaplan–Meier method was employed to examine the relationship between a new index (combining TBS and AAPR) and long‐term outcomes. The predictive efficacy of this index was compared to other conventional indicators. A total of 560 patients were included in the study. Based on TBS and AAPR stratification, patients were classified into three groups. Kaplan–Meier curves demonstrated that 124 patients with low TBS and high AAPR had the best overall survival (OS) and recurrence‐free survival (RFS), while 170 patients with high TBS and low AAPR had the worst outcomes (log‐rank *p* < 0.001). Multivariate analyses identified the combined index as an independent predictor of OS and RFS. Furthermore, the index showed superior accuracy in predicting OS and RFS compared to other conventional indicators. Collectively, this study demonstrated that the combination of liver function and tumour morphology provides a synergistic effect in evaluating the prognosis of ICC patients. The novel index combining TBS and AAPR effectively stratified postoperative survival outcomes in ICC patients undergoing curative resection.

## 1 INTRODUCTION

Primary liver cancer primarily consists of hepatocellular carcinoma (HCC), intrahepatic cholangiocarcinoma (ICC) and combined hepatocellular‐cholangiocarcinoma (cHCC‐CCA).^1^ ICC is the second most common primary hepatic malignancy, accounting for 15%–20% of newly diagnosed liver malignancies.^2^ Most patients are diagnosed at advanced stages, losing the opportunity for curative resection.^3^ Even for those undergoing surgical treatment, the 5‐year survival rate remains dismal.^4^ The poor long‐term survival rate of ICC may be related to the high tendency for regional and distant metastasis and the lack of effective systemic treatment options. Therefore, improving the prognosis prediction for ICC patients may be an effective strategy to address its poor outcomes.^5^ While numerous biomarkers have been reported to predict the prognosis of ICC patients, their clinical application remains limited. There is an urgent need for a simple and effective marker to better predict the prognosis of ICC patients, enabling clinicians to implement personalized treatment early to improve patient survival.

The tumour burden score (TBS), a metric based on tumour size and number, was first proposed in 2017 by Sasaki et al. as a survival prediction tool for patients undergoing hepatic resection for colorectal liver metastasis (CRLM).^6^ Compared with tumour size and number, TBS showed better efficacy in predicting long‐term survival. Our previous study demonstrated that the prognosis of ICC patients could be stratified by TBS, with elevated TBS associated with decreased overall survival (OS) and recurrence‐free survival (RFS).^7^ In addition to tumour burden, liver function indicators such as the Child‐Pugh grade, albumin‐bilirubin (ALBI) grade and albumin‐to‐alkaline phosphatase ratio (AAPR) have been reported to evaluate postoperative complications and prognosis in patients undergoing liver resection.^8^, ^9^ In our previous work, we compared the accuracy of these three indices in predicting long‐term survival of ICC patients undergoing hepatectomy and found that both the ALBI grade and AAPR were useful surrogate markers for identifying patients at risk of poor postoperative outcomes. Furthermore, AAPR exhibited better effectiveness.

Given that both liver function and tumour burden show promising performance in predicting outcomes of ICC patients, we hypothesized that a combined index would demonstrate even better predictive ability. Thus, in this study, we propose a novel index combining TBS and AAPR to evaluate its effect on OS and RFS in ICC patients. Additionally, we compare its predictive efficacy with other conventional indicators.

## 2 METHODS

### 2.1 Study population and data

This retrospective analysis was conducted in accordance with the Declaration of Helsinki^10^ and utilized data from Chongqing University Cancer Hospital (Chongqing, China), West China Hospital (Sichuan, China) and The Second Affiliated Hospital of Guangzhou University of Chinese Medicine (Guangdong, China). The study was approved by the Ethics Committees of these institutions. All the pathologically diagnosed ICC patients who underwent curative surgery at these institutions from 2011 to 2018 were initially evaluated. Clinicopathological characteristics and postoperative outcomes were reviewed to select candidates. Exclusion criteria included patients with extrahepatic metastasis or other malignancies, patients with a positive surgical margin, local organ invasion. In addition, 11 patients loss of follow‐up and 23 patients with incomplete data on variables of interest (such as CA19‐9 levels, number of lesions, albumin value and alkaline phosphatase value) were also excluded. All patients or their relatives provided informed consent.

### 2.2 Variables and outcomes of interest

The demographic and clinicopathological parameters included gender, age, hepatitis B virus positivity (HBsAg), presence of intrahepatic bile duct stones, presence of liver cirrhosis, morphologic type (MF, mass forming; IG, intraductal growth; PI, periductal infiltrating), CA19‐9 (U/mL), maximum lesion size (cm), number of lesions, albumin (g/dL), alkaline phosphatase, differentiation, liver capsule invasion status, perineural invasion status, major vascular invasion status, microvascular invasion status and TNM stages according to the American Joint Committee on Cancer (AJCC) 8th edition staging. Microvascular invasion was defined as involvement of hepatic parenchymal vessels found on histological examination. Major vascular invasion was defined as invasion of the main or first and second‐order branches of the portal vein or hepatic artery.^11^

As previously reported, TBS was defined as the distance from the origin of the Cartesian plane, composed of the maximum tumour size (*x*‐axis) and the number of tumours (*y*‐axis). Thus, TBS^2^ = (maximum tumour diameter)^2^ + (number of tumours)^2^. The cut‐off value of TBS was consistent with our previous study,^7^ a value of 4.71 was used to divided patients into high‐ or low‐TBS groups. AAPR was calculated as the ratio of albumin to alkaline phosphatase; a value of 0.348, as previously reported, was used to stratify patients.^12^ By combining the optimal cut‐off values of AAPR and TBS, patients were divided into three subgroups for comparative analysis: low TBS combined with high AAPR, high TBS combined with low AAPR and low TBS combined with low AAPR or high TBS combined with high AAPR.

Patients were followed up according to the National Comprehensive Cancer Network guidelines, with monthly routine tumour marker and imaging examinations (such as ultrasound, computed tomography and/or magnetic resonance imaging) in the first 6 months to monitor recurrence, followed by examinations every 3 months, and then every 6 months thereafter. Those who did not return to the hospital for re‐examination were followed up by telephone survey. OS was calculated from liver resection to death or last follow‐up. RFS was defined as the time from the first surgery to the earliest evidence of recurrence or last follow‐up.

### 2.3 Statistical analysis

Continuous variables were reported as median and interquartile range (IQR), while categorical variables were reported as counts and percentages (%). Chi‐squared tests were used to compare categorical variables, while Kruskal–Wallis one‐way analysis of variance was used to compare continuous variables. Kaplan–Meier curves were used to describe patient survival, with differences tested using the log‐rank test. Univariate and multivariate Cox regression models were used to identify independent prognostic risk factors. In the univariate analysis, clinical and pathological parameters with *p* < 0.05 were selected for multivariate analysis. All data were analysed using SPSS (version 23.0, IBM Corp., Armonk, NY, USA) and MedCalc (version 20.0.3.0, Ostend, Belgium). A *p*‐value less than 0.05 was considered statistically significant.

## 3 RESULTS

### 3.1 Patient characteristics

A total of 560 ICC patients who underwent curative resection were included in this study (Table 1). Among them, 286 were male (51.1%) and 274 were female (48.9%). The median age was 59.0 years (IQR: 50.0–65.0). The majority were HBsAg‐negative (397 cases, 70.9%). Intrahepatic bile duct stones were absent in 464 patients (82.9%), while 96 patients had stones. Liver cirrhosis was present in 137 cases (24.5%) and absent in 423 cases (75.5%). The morphological type was determined for most patients, with 445 (79.4%) having mass‐forming (MF) or intraductal growth (IG) types, 100 patients having periductal infiltrating (PI) or MF + PI types, and only 10 patients with unknown morphology. Low‐grade differentiation was observed in 15 cases (2.7%), with the remaining 545 cases (97.3%) being of moderate to poor differentiation.

**TABLE 1:** Characteristics of included patients with intrahepatic cholangiocarcinoma.

Variable	Total (*n* = 560)
Gender, *n* (%)	
Male	286 (51.1)
Female	274 (48.9)
Age, year, median (IQR)	59 (50–65)
HBsAg, *n* (%)	
Positive	163 (29.1)
Negative	397 (70.9)
Hepatolithiasis, *n* (%)	
Present	96 (17.1)
Absent	464 (82.9)
Cirrhosis, *n* (%)	
Present	137 (24.5)
Absent	423 (75.5)
Morphologic type	
MF, IG	445 (79.4)
PI, MF + PI	100 (17.9)
Unknown	15 (2.7)
CA19‐9, U/mL, median (IQR)	71.9 (19.1–813.7)
Size of largest lesion, cm, median (IQR)	5.7 (4.2–7.9)
Number of lesions, median (IQR)	1 (1–2)
TBS, median (IQR)	5.88 (4.41–7.96)
Albumin, g/dL, median (IQR)	42.8 (39.4–45.0)
ALP, U/L, median (IQR)	108 (85–157)
AAPR, median (IQR)	0.387 (0.273–0.514)
Differentiation, *n* (%)	
Well	15 (2.7)
Moderate to poor	545 (97.3)
Liver capsule invasion, *n* (%)	
Present	367 (65.5)
Absent	193 (34.5)
Perineural invasion, *n* (%)	
Present	86 (15.4)
Absent	474 (84.6)
Major vascular invasion, *n* (%)	
Present	146 (26.1)
Absent	414 (73.9)
Microvascular invasion, *n* (%)	
Present	65 (11.6)
Absent	495 (88.4)
Lymph node invasion, *n* (%)	
Present	131 (23.4)
Absent	429 (76.6)
AJCC 8th edition stage, *n* (%)	
I	96 (17.1)
II	56 (10.0)
III	408 (72.9)

Abbreviations: AAPR, albumin‐to‐alkaline phosphatase ratio; AJCC, American Joint Committee on Cancer; ALP, alkaline phosphatase; CA19‐9, carbohydrate antigen 19‐9; IG, intraductal growth; IQR, interquartile range; MF, mass‐forming; PI, periductal infiltrating; TBS, tumour burden score.

Preoperative levels of CA19‐9, albumin and alkaline phosphatase were 71.9 U/mL (IQR: 19.1–813.7), 42.8 g/dL (IQR: 39.4–45.0) and 108 U/L (IQR: 85–157), respectively. The maximum lesion size was 5.7 cm (IQR: 4.2–7.9), and the median lesion number was 1 (IQR: 1–2). The TBS and AAPR had median values of 5.88 (IQR: 4.41–7.96) and 0.387 (IQR: 0.273–0.514), respectively. According to the AJCC 8th edition staging, 96 patients (17.1%) were classified as stage I, 56 (10.0%) as Stage II, and 408 (72.9%) as Stage III. Tumour invasion indicators included liver capsule invasion (367 cases, 65.5%), perineural invasion (86 cases, 15.4%), major vascular invasion (146 cases, 26.1%), microvascular invasion (65 cases, 11.6%) and lymph node invasion (131 cases, 23.4%).

### 3.2 Association between the index and clinicopathological feature

As summarized in Table 2, 124 patients (22.1%) were in the low TBS and high AAPR group, 266 patients (47.5%) were in the low TBS and low AAPR/high TBS and high AAPR group, and 170 patients (30.4%) were in the high TBS and low AAPR group. The highest CA19‐9 levels were observed in the high TBS and low AAPR group (177.2, IQR: 24.9–1000.0), while the levels in the other groups were 34.2 (low TBS and high AAPR, IQR: 13.4–755.4) and 80.6 (low TBS and low AAPR/high TBS and high AAPR, IQR: 21.5–805.3), respectively, though this was not statistically significant. The high TBS and low AAPR group had the largest median maximum lesion size (7.9, IQR: 6.0–9.6). The low TBS and low AAPR / high TBS and high AAPR group had the highest proportion of single lesions (187, 70.3%), while the high TBS and low AAPR group had the highest proportion of multiple lesions (60%, 35.3%).

**TABLE 2:** Characteristics stratified by TBS and AAPR grade.

Variable	Low TBS and high AAPR	Low TBS and low AAPR/high TBS and high AAPR	High TBS and low AAPR	*p‐*value
Cases, *n* (%)	124 (22.1)	266 (47.5)	170 (30.4)	
Gender, *n* (%)				0.885
Male	65 (52.4)	133 (50.0)	88 (51.8)	
Female	59 (47.6)	133 (50.0)	82 (48.2)	
Age, year, median (IQR)	60 (52–68)	58 (49–71)	59 (51–68)	0.082
HBsAg, *n* (%)				0.085
Positive	40 (32.3)	85 (32.0)	38 (22.4)	
Negative	84 (67.7)	181 (68.0)	132 (77.6)	
Hepatolithiasis, *n* (%)				0.189
Present	28 (22.6)	41 (15.4)	27 (15.9)	
Absent	96 (77.4)	225 (84.6)	143 (84.1)	
Cirrhosis, *n* (%)				0.085
Present	38 (30.6)	66 (24.8)	33 (19.4)	
Absent	86 (69.4)	200 (75.2)	137 (80.6)	
Morphologic type				0.921
MF, IG	98 (79.0)	213 (80.1)	134 (78.9)	
PI, MF + PI	25 (20.2)	44 (16.5)	31 (18.2)	
Unknown	1 (0.8)	9 (3.4)	5 (2.9)	
CA19‐9, U/mL, median (IQR)	34.2 (13.4–755.4)	80.6 (21.5–805.3)	177.2 (24.9–1000.0)	0.139
Size of largest lesion, cm, median (IQR)	3.5 (2.6–4.2)	6.0 (5.0–7.3)	7.9 (6.0–9.6)	<0.001
Tumour number, *n* (%)				<0.001
Solitary	108 (87.1)	187 (70.3)	110 (64.7)	
Multiple	16 (12.9)	79 (29.7)	60 (35.3)	
Number of lesions, median (IQR)				1 (1–2)
TBS, median (IQR)	3.64 (2.79–4.14)	6.08 (5.09–7.47)	7.96 (6.08–9.82)	<0.001
Albumin, g/dL, median (IQR)	43.2 (41.0–45.6)	43.7 (40.7–45.9)	41.1 (37.9–43.9)	0.155
ALP, U/L, median (IQR)	85 (71–97)	99 (82–122)	166 (136–222)	<0.001
AAPR, median (IQR)	0.523 (0.435–0.626)	0.436 (0.359–0.538)	0.252 (0.182–0.304)	<0.001
Differentiation, *n* (%)				0.869
Well	2 (1.6)	8 (3.0)	5 (2.9)	
Moderate to poor	122 (98.4)	258 (97.0)	165 (97.1)	
Liver capsule invasion, *n* (%)				<0.001
Present	63 (50.8)	194 (72.9)	110 (64.7)	
Absent	61 (49.2)	72 (27.1)	60 (35.3)	
Perineural invasion, *n* (%)				0.999
Present	19 (15.3)	41 (15.4)	26 (15.3)	
Absent	105 (84.7)	225 (84.6)	144 (84.7)	
Major vascular invasion, *n* (%)				<0.001
Present	15 (12.1)	71 (26.7)	60 (35.3)	
Absent	109 (87.9)	195 (73.3)	110 (64.7)	
Microvascular invasion, *n* (%)				0.177
Present	11 (8.9)	28 (10.5)	26 (15.3)	
Absent	113 (91.1)	238 (89.5)	144 (84)	
Lymph node invasion, *n* (%)				<0.001
Present	15 (12.1)	60 (22.6)	56 (32.9)	
Absent	109 (87.9)	206 (77.4)	114 (67.1)	
AJCC 8th edition stage, *n* (%)				<0.001
I	47 (37.9)	31 (11.7)	18 (10.6)	
II	8 (6.5)	25 (9.4)	23 (13.5)	
III	69 (55.6)	210 (78.9)	129 (75.9)	

Abbreviations: AAPR, albumin‐to‐alkaline phosphatase ratio; ALP, alkaline phosphatase; AJCC, American Joint Committee on Cancer; CA19‐9, carbohydrate antigen 19‐9; IG, intraductal growth; IQR, interquartile range; MF, mass forming; PI, periductal infiltrating; TBS, tumour burden score.

In addition, patients in the high TBS and low AAPR group were more frequently associated with liver capsule invasion, major vascular invasion, lymph node invasion, and advanced TNM stages (all *p* < 0.001).

### 3.3 Impact of the index on prognosis

**FIGURE 1:**
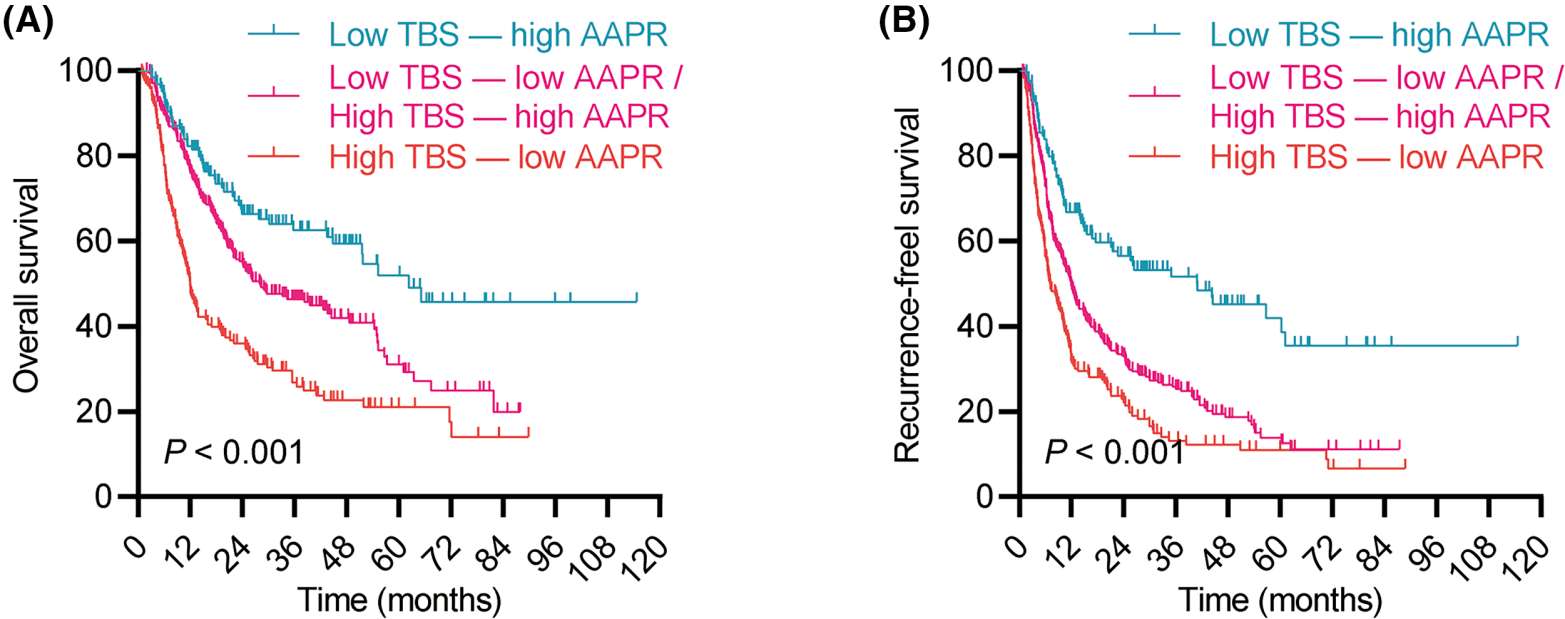
Kaplan–Meier curves demonstrating differences in overall survival (OS) (A) and recurrence‐free survival (RFS) (B) among patients stratified by tumour burden score (TBS) and albumin‐to‐alkaline phosphatase ratio (AAPR). Groups include low TBS/high AAPR, low TBS/low AAPR, high TBS/high AAPR and high TBS/low AAPR.

Kaplan–Meier curves indicated that the index was a strong predictor of long‐term outcomes in ICC patients. Patients with low TBS and high AAPR had the best OS and RFS, while those with high TBS and low AAPR had the worst OS and RFS (Figure 1).

Cox regression analyses identified several factors affecting postoperative OS and RFS in ICC patients (Table 3). Hepatolithiasis, CA19‐9 level, tumour size, tumour number, tumour differentiation, major vascular invasion, MVI, TNM stage and the combined TBS and AAPR index were potential factors influencing OS. Multivariate analysis confirmed that elevated CA19‐9 level, multiple tumours, presence of MVI, lymph node invasion and the combined TBS and AAPR groups (low TBS and low AAPR/high TBS and high AAPR, high TBS and low AAPR) were independent risk factors for poor OS. For RFS, similar factors were identified, including elevated CA19‐9 level, multiple tumours, presence of MVI, major vascular invasion, lymph node invasion and the combined TBS and AAPR groups.

**TABLE 3:** Identification of prognostic factors for overall survival and recurrence‐free survival.

Variables	Overall survival				Recurrence‐free survival			
	Univariate		Multivariate		Univariate		Multivariate	
	HR (95% CI)	*p‐*value	HR (95% CI)	*p*‐value	HR (95% CI)	*p‐*value	HR (95% CI)	*p‐*value
Gender (F/M)	0.871 (0.701–1.084)	0.216			0.957 (0.790–1.159)	0.655		
Age	0.996 (0.986–1.007)	0.465			0.969 (0.916–1.026)	0.286		
HBsAg	1.183 (0.934–1.499)	0.164			1.193 (0.968–1.470)	0.098		
Hepatolithiasis	1.444 (1.103–1.889)	0.008	1.286 (0.969–1.706)	0.081	1.001 (0.774–1.294)	0.996		
Cirrhosis	1.175 (0.915–1.510)	0.206			1.091 (0.872–1.365)	0.447		
Morphologic type (PI, MF + PI/MF, IG)	1.151 (0.914–1.449)	0.233			1.077 (0.878–1.321)	0.475		
CA19‐9 (≥37/≤37)	1.926 (1.639–2.264)	<0.001	1.618 (1.366–1.916)	<0.001	1.536 (1.335–1.767)	<0.001	1.423 (1.234–1.641)	<0.001
Tumour size (≥5/<5)	1.317 (1.053–1.647)	0.016	0.883 (0.665–1.171)	0.387	1.499 (1.229–1.829)	0.001	0.952 (0.741–1.223)	0.701
Tumour number (multiple/solitary)	1.678 (1.334–2.113)	<0.001	1.465 (1.151–1.866)	0.002	1.711 (1.393–2.103)	<0.001	1.508 (1.218–1.868)	<0.001
Differentiation (moderate to poor/well)	1.862 (1.448–2.394)	<0.001	1.238 (0.897–1.710)	0.195	1.703 (1.373–2.113)	<0.001	0.981 (0.780–1.233)	0.868
Liver capsule invasion	1.096 (0.869–1.382)	0.439			1.229 (0.992–1.512)	0.051		
Perineural invasion	1.222 (0.958–1.558)	0.106			1.389 (1.073–1.799)	0.013	1.097 (0.828–1.454)	0.517
Major vascular invasion	1.762 (1.329–2.335)	0.001	0.927 (0.717–1.198)	0.562	1.335 (1.074–1.658)	0.009	1.543 (1.235–1.928)	<0.001
MVI	1.668 (1.227–2.269)	0.001	1.823 (1.402–2.371)	<0.001	1.946 (1.477–2.562)	<0.001	1.671 (1.248–2.236)	<0.001
Lymph node invasion	2.374 (1.875–3.004)	<0.001	1.627 (1.249–2.121)	<0.001	1.914 (1.544–2.373)	<0.001	1.484 (1.167–1.886)	0.001
AJCC 8th edition stage (III/I–II)	1.300 (1.114–1.518)	0.001	1.035 (0.871–1.231)	0.694	1.329 (1.160–1.524)	<0.001	1.104 (0.951–1.282)	0.194
Combined TBS and AAPR								
Low TBS and high AAPR	Ref.		Ref.		Ref.			
Low TBS and low AAPR/high TBS and high AAPR	1.615 (1.168–2.233)	<0.001	1.404 (1.004–1.963)	0.047	1.971 (1.487–2.614)	<0.001	1.691 (1.211–2.361)	0.002
High TBS and low AAPR	2.917 (2.095–4.062)	<0.001	2.151 (1.518–3.051)	<0.001	2.806 (2.084–3.779)	<0.001	2.180 (1.511–3.145)	<0.001

Abbreviations: AAPR, albumin‐to‐alkaline phosphatase ratio; CA19‐9, carbohydrate antigen 19‐9; CI, confidence interval; F, female; HR, hazard ratio; IG, intraductal growth; M, male; MF, mass‐forming; MVI, microvascular invasion; PI, periductal infiltrating; Ref., reference; TBS, tumour burden score.

**FIGURE 2:**
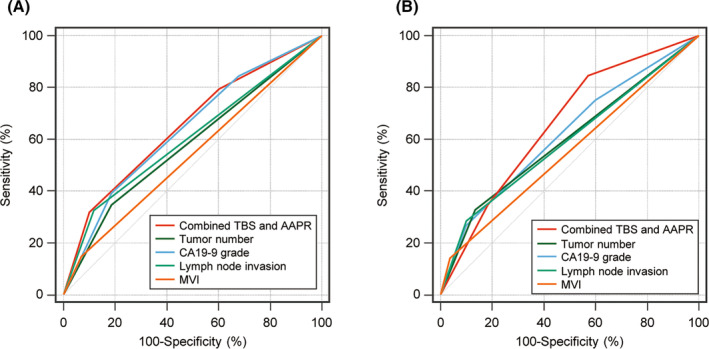
Comparison of AUC values for combined TBS and AAPR, tumour number, CA19‐9, lymph node invasion and microvascular invasion in predicting OS (A) and RFS (B). AUC, area under the receiver operating characteristic curve; AAPR, albumin‐to‐alkaline phosphatase ratio; CA19‐9, carbohydrate antigen 19‐9; MVI, microvascular invasion; TBS, tumour burden score.

ROC curves compared the discriminatory power of these independent risk factors in predicting prognosis (Figure 2). The combined TBS and AAPR index demonstrated superior predictive accuracy for OS and RFS compared to other factors, with AUC values of 0.653 for OS and 0.658 for RFS. These results suggest that the combined TBS and AAPR index is a promising predictive indicator compared with conventional markers.

## 4 DISCUSSION

Patients with ICC are usually asymptomatic, leading to a delay in disease diagnosis and worse patient prognosis.^13^ The diagnosis of ICC is always adequate by using blood workup, imaging and tissue biopsy. Patients with ICC are often associated with abnormal liver function, such as elevated gamma‐glutamyl transpeptidase and alkaline phosphatase levels.^14^ CA19‐9 and carcinoembryonic antigen are the most commonly used tumour markers for ICC. For imaging, computed tomography (CT) and contrast‐enhanced magnetic resonance imaging (MRI) scan have relatively specific performance and show comparable capability in detecting primary and satellite lesions.^15^ Apart from bloodwork, and imaging findings, pathologic confirmation is usually necessary before initiating systemic therapy as well as after undergoing curative resection. Liquid biopsy is not the routine test for the diagnosis of ICC, but the detection of circulating tumour deoxyribonucleic acid (ctDNA) and whole‐exome sequencing of peripheral blood are helpful in assessment of therapeutic effect.^16^

Over the past few decades, the incidence of ICC has steadily increased, with typically low 5‐year survival rates following surgery. However, significant differences in long‐term prognosis exist.^17^, ^18^ Therefore, stratifying and analysing patients' prognoses can help make appropriate medical decisions and improve long‐term outcomes. Both TBS and AAPR, common indicators in cancer patients, have predictive effects on the prognosis of ICC patients.^7^, ^19^ Our study combines TBS and AAPR to predict ICC patients' prognosis, exploring their combined role in predicting long‐term outcomes after curative surgery. This is the first study to investigate the role of TBS combined with AAPR in the postoperative prognosis of ICC patients, holding important clinical implications.

TBS is a comprehensive morphometric measurement based on tumour size and number, showing good predictive effects in HCC, lung cancer and colorectal cancer.^20^, ^21^, ^22^, ^23^ For instance, Zorays Moazzam et al. indicated that TBS is independently associated with a higher risk of recurrence, and the tumour burden score calculated using hazard ratios and multivariate analysis more accurately predicts recurrence risk, time and pattern of HCC than traditional clinicopathological factors. Moreover, the risk of recurrence varies according to the TBS of HCC patients; those with low TBS are more prone to intrahepatic recurrence, while those with high TBS are more likely to experience extrahepatic recurrence.^24^ Our study also found that increasing TBS levels correlate with worse postoperative long‐term outcomes in ICC patients. Additionally, previous research has shown a strong nonlinear association between TBS after curative resection of ICC and survival rates, suggesting TBS as a prognostic factor.^25^ The impact of surgical margin status on overall survival is influenced by tumour status, with increased margin width associated with improved 5‐year OS in patients with low or moderate TBS.^26^

AAPR, a blood biomarker, has been widely studied in oncology, especially in prognostic analysis of HCC.^27^ Studies have found that preoperative AAPR levels are associated with HCC prognosis after curative resection.^28^ AAPR can be used for routine preoperative testing, crucial for early detection of high‐risk patients and personalized adjuvant therapy. Our study found that decreased AAPR levels are associated with worse postoperative prognosis in ICC patients. Furthermore, in liver transplant recipients with hepatocellular carcinoma, decreased AAPR levels are associated with poorer prognosis, highlighting the value of AAPR as a prognostic indicator.^29^ A meta‐analysis summarized that low AAPR predicts poorer OS and RFS in HCC patients, consistent with our results.^30^ Our previous study demonstrated that AAPR was more accurate in evaluating OS of ICC patients compared to Child–Pugh score and ALBI grade (C index: 0.6000 vs. 0.559 vs. 0.528).^8^ Our study shows that combining TBS and AAPR performs better in postoperative prediction for ICC patients (OS accuracy: 0.653, 95% CI: 0.611–0.692; RFS accuracy: 0.658, 95% CI: 0.617–0.698).

The combination of TBS and AAPR as predictive factors to assess postoperative OS and RFS of ICC patients is relatively underexplored but holds significant value. TBS is a scoring system for tumour morphology and combining it with blood biomarkers may yield higher accuracy.^31^ Previous studies have combined TBS with CA19‐9 to evaluate long‐term outcomes after curative resection in ICC patients, suggesting that low TBS/low CA19‐9 patients have the best OS and RFS, while high TBS/high CA19‐9 patients have the worst outcomes.^7^ Additionally, Zorays Moazzam et al. also conducted related research on TBS combined with CA19‐9. They found that despite differences in prediction between TBS and CA19‐9, using TBS combined with CA19‐9 is still an independent factor for predicting OS in ICC patients. Furthermore, they suggested that the interaction between tumour morphology and biology determines the long‐term prognosis after hepatic resection for intrahepatic cholangiocarcinoma. Prognostic models combining TBS with CA19‐9 can provide information for prognosis discussions and help identify which ICC patients may benefit more from adjuvant chemotherapy rather than upfront surgery.^32^ Therefore, our study suggests that combining TBS with AAPR for postoperative outcome estimation has significant clinical implications, showing clear prognostic differences between stratified groups and providing personalized treatment recommendations.

Despite limitations such as a relatively small sample size and the need for further validation, our research holds significant clinical value. Compared to studies solely using TBS, our analysis includes blood biomarker indicators, providing a more comprehensive postoperative evaluation and reference for personalized treatment decisions. As a multicentre study, our research benefits from high data quality and diverse patient samples. This study is the first to evaluate ICC patients' prognosis using TBS combined with AAPR, highlighting its importance for survival analysis and prognosis assessment. Future studies should further compare the predictive effects of TBS combined with AAPR against other factors, such as TBS combined with ALBI or CA19‐9.

## 5 CONCLUSIONS

The combination of TBS and AAPR demonstrates significant predictive efficacy in assessing ICC prognosis, especially for predicting postoperative recurrence. We suggest that surgeons, oncologists and pathologists consider using TBS combined with AAPR to provide more efficient and rational clinical recommendations for ICC patients undergoing curative surgery.

## AUTHOR CONTRIBUTIONS

**Sheng Wang:** Data curation (equal); formal analysis (equal); methodology (equal); software (equal); writing – original draft (equal). **Maoyun Liu:** Data curation (equal); writing – original draft (equal). **Lei Xiang:** Data curation (equal); formal analysis (equal); software (equal); writing – original draft (equal). **Haizhou Qiu:** Data curation (equal); formal analysis (equal); methodology (equal); writing – original draft (equal). **Luo Cheng:** Formal analysis (equal); methodology (equal); software (equal). **Zuotian Huang:** Formal analysis (equal); methodology (equal). **Tao Wen:** Formal analysis (equal). **Wenyuan Xie:** Software (equal). **Sipeng Li:** Methodology (equal). **Cheng Zhang:** Methodology (equal); software (equal). **Genshu Wang:** Supervision (equal); writing – original draft (equal). **Hui Li:** Conceptualization (lead); data curation (equal); formal analysis (equal); funding acquisition (equal); supervision (equal); writing – original draft (equal); writing – review and editing (equal). **Dewei Li:** Conceptualization (equal); funding acquisition (equal); supervision (equal); writing – original draft (equal); writing – review and editing (equal).

## FUNDING INFORMATION

This work was supported by grants from the Natural Science Foundation of China (82203823), the China Postdoctoral Science Foundation (2023 M730436, 2022TQ0393), the Chongqing Postdoctoral Science Foundation (2022CQBSHTB2029), Chongqing medical scientific research project (Joint project of Chongqing Health Commission and Science and Technology Bureau) (2023MSXM104, 2024ZDXM008) and the Natural Science Foundation of Chongqing (CSTB2022NSCQ‐MSX0477, CSTB2022NSCQ‐MSX1174).

## CONFLICT OF INTEREST STATEMENT

The authors declare no potential conflicts of interest.

## Supporting information


**Table S1:** Comparison of predictive value in OS and RFS.

## Data Availability

All data included in this study are available upon request by contact with the corresponding author.
