# Supplementary material for: Tumour burden score combined with albumin‐to‐alkaline phosphatase ratio predicts prognosis in patients with intrahepatic cholangiocarcinoma
Source: J Cell Mol Med. 2024 Jul 3;28(13):e18530. doi: 10.1111/jcmm.18530 (PMC11222657; doi:10.1111/jcmm.18530)
Supplement: Supplementary file 1 — Table S1: Comparison of predictive value in OS and RFS. [file JCMM-28-e18530-s001.docx]

Supplementary table 1. Comparison of predictive value in OS and RFS.

|  | Accuracy | 95% CI | P-value |
| --- | --- | --- | --- |
| OS |  |  |  |
| -Combined TBS and AAPR | 0.653 | 0.611-0.692 | Ref. |
| -Tumor number | 0.581 | 0.538-0.622 | 0.013 |
| -CA19-9 grade | 0.638 | 0.596-0.678 | 0.595 |
| -Lymph node invasion | 0.604 | 0.562-0.645 | 0.054 |
| -MVI | 0.539 | 0.496-0.581 | <0.001 |
| RFS |  |  |  |
| -Combined TBS and AAPR | 0.658 | 0.617-0.698 | Ref. |
| -Tumor number | 0.597 | 0.554-0.638 | 0.029 |
| -CA19-9 grade | 0.620 | 0.578-0.660 | 0.215 |
| -Lymph node invasion | 0.592 | 0.550-0.634 | 0.022 |
| -MVI | 0.553 | 0.510-0.595 | <0.001 |

OS, overall survival; RFS, recurrence-free survival; CA19-9, carbohydrate antigen 19-9; TBS, tumor burden score; AAPR, albumin-to-alkaline phosphatase ratio; MVI, microvascular invasion.
